# L-Lactate Protects Skin Fibroblasts against Aging-Associated Mitochondrial Dysfunction *via* Mitohormesis

**DOI:** 10.1155/2015/351698

**Published:** 2015-06-10

**Authors:** Jaroslav Zelenka, Aleš Dvořák, Lukáš Alán

**Affiliations:** ^1^Institute of Physiology, Czech Academy of Sciences, Vídeňská 1083, 142 20 Prague, Czech Republic; ^2^Department of Biochemistry and Microbiology, Institute of Chemical Technology Prague, Technická 5, 166 28 Prague, Czech Republic

## Abstract

A moderate elevation of reactive oxygen species (ROS) production and a mild inhibition of mitochondrial respiratory chain have been associated with a health promotion and a lifespan extension in several animal models of aging. Here, we tested whether this phenomenon called mitohormesis could be mediated by L-lactate. The treatment with 5 mM L-lactate significantly increased H_2_O_2_ production and slightly inhibited the respiration in cultured skin fibroblasts and in isolated mitochondria. The L-lactate exposure was associated with oxidation of intracellular glutathione, phosphorylation of 5′AMP-activated protein kinase (AMPK), and induction of peroxisome proliferator-activated receptor gamma coactivator 1*α* (PGC1*α*) transcription. A replicative aging of fibroblasts (L0) with a constant (LC), or intermittent 5 mM L-lactate (LI) in media showed that the high-passage LI fibroblasts have higher respiration, lower H_2_O_2_ release, and lower secretion of L-lactate compared to L0 and LC. This protection against mitochondrial dysfunction in LI cells was associated with lower activity of mechanistic target of rapamycin complex 1 (mTORC1), less signs of cellular senescence, and increased autophagy compared to L0 and LC. In conclusion, we demonstrated that intermittent but not constant exposure to L-lactate triggers mitohormesis, prevents aging-associated mitochondrial dysfunction, and improves other markers of aging.

## 1. Introduction

Aging and associated pathologies represent the major global health problem of the 21st century [1]. A development of mitochondrial dysfunction and an accumulation of oxidative damage belong to the hallmarks of aging. This led to a suggestion of the “mitochondrial free radical theory of aging” (MFRTA) which states that aging results from accumulation of oxidative damage caused by mitochondrial reactive oxygen species (ROS). This damage could be further amplified by ROS-derived mutations in mitochondrial DNA (mtDNA) resulting in the increased ROS leak from invalid respiratory chain [2]. This theory suggests that increasing antioxidant protection could attenuate the rate of aging by limiting the detrimental effects of ROS to the organism. However, it has been recently reported that manipulation of antioxidant protection has little or no effect on aging, suggesting that MFRTA concept needs to be revisited [3–5]. ROS not only are damaging in high concentrations, but also serve as signaling molecules under physiological conditions. Moreover, moderate elevation of ROS levels could even protect against oxidative damage through induction of antioxidant and detoxification enzymes [6]. Recently, the extended lifespan and the prevention of chronic diseases has been described in the group of genetic and pharmaceutical models ranging from yeasts to mice, which share a phenotype of mild inhibition of mitochondrial respiratory chain accompanied with a moderate elevation of mitochondrial ROS production. This effect termed the mitochondrial hormesis or mitohormesis [4–8] has been linked to the activation of protective and quality control mechanisms including hypoxia-inducible factor 1*α* (HIF1*α*), the 5′AMP-activated protein kinase (AMPK), and the unfolded protein response (UPR) signaling [7–10]. Moreover, mitohormesis has been linked to the beneficial effects of regular physical exercise, the intervention associated with the intermittent elevation of tissue and serum l-lactate [8, 11]. l-lactate is an endogenous metabolite which accumulates during bioenergetic stress and has long been viewed as just a potentially harmful metabolic byproduct. However, recent evidence suggests that l-lactate has also an important role in cell signaling. It binds and activates a Gi-coupled receptor GPR81, causing a reduced lipolysis in adipocytes [12], an increase in proliferation and metastasis of tumor cells [13], and an attenuation of innate immune response [14]. In brain, l-lactate serves as a humoral transmitter [15] presumably also through GPR81 signaling [16]. In addition, l-lactate treatment has been linked to a rise in intracellular ROS levels [17], an activation of mitochondrial biogenesis through upregulation of the peroxisome proliferator-activated receptor gamma coactivator 1*α* (PGC1*α*) [13, 17–19], an elevation of the brain-derived neurotrophic factor (BDNF) levels [20, 21], a stabilization of HIF1*α* [22], and an activation of the transcription factor nuclear factor *κ*B (NF*κ*B) [17, 23]. A chronic elevation of serum and tissue l-lactate which occurs in subjects with the metabolic syndrome has been linked to the activation of inflammatory pathways through HIF1*α* and NF*κ*B signaling [23]. Moreover, a high l-lactate in tumor microenvironment serves as a metabolic fuel for the so-called oxidative cancer cells rich in mitochondria and promotes their proliferation and metastases through PGC1*α* induction [13, 22, 24]. On the contrary, little is known about signaling effects of the intermittent l-lactate elevation associated with the exercise.

Here, we aimed at investigating whether the treatment of primary fibroblasts with l-lactate levels common during exercise could trigger the signaling pathways involved in mitohormesis and whether the intermittent l-lactate elevation could attenuate the signs of cellular and mitochondrial aging of primary skin fibroblasts in the culture.

## 2. Materials and Methods

### 2.1. Animals

Wistar rats (3 months old, 250 g, housed in Institute of Physiology) were sacrificed with CO_2_ and their livers were quickly excised and subjected to isolation of mitochondria. A tissue graft for establishment of a primary skin fibroblast culture was taken from the external ear. The local animal research committee approved the protocols for all aspects of the animal studies in accordance with the Guide for the Care and Use of Laboratory Animals, as adopted and promulgated by the United States National Institutes of Health.

### 2.2. Cell Culture

The rat skin fibroblasts were cultured in the Dulbecco modified Eagle medium with 5 mM glucose, 3 mM glutamine, and 10% fetal bovine serum in the atmosphere containing 5% CO_2_. Experimental media contained 5 mM l-lactate prepared from a neutralized potassium l-lactate (Purasal HiPure P Plus, Corbion, Amsterdam, Netherlands). Fibroblasts were split twice a week before reaching 90% density and seeded at a constant 20% density. The LC cells were cultured continuously in the medium containing 5 mM l-lactate. The LP cells received 5 mM l-lactate 12 hours before each passage. The proportion of senescent fibroblasts was estimated as a percent of positive cells after staining for senescence-associated *β*-galactosidase with X-Gal (Sigma-Aldrich, St. Louis, MO, USA) as described in [25]. MTT assay was performed as described previously [26].

### 2.3. Preparation of Liver and Fibroblast Mitochondria

Liver tissue (10 g) or cultured fibroblasts (10^7^ cells, collected from 600 cm^2^ of confluent culture plates) were homogenized in 180 mM KCl, 5 mM potassium-MOPS, 5 mM potassium-EGTA, pH 7.2 with a Potter-Elvehjem tissue grinder. Samples were then purified by a sequential centrifugation at 500 ×g for 5 minutes and 3000 ×g for 5 minutes to get rid of a cell debris and peroxisomes. Mitochondria were collected by a centrifugation at 8000 ×g for 10 minutes. Mitochondrial samples were diluted according to protein concentration to 10 mg/mL of the isolation buffer, stored on ice, and immediately proceeded to respirometric and H_2_O_2_ assay. The quality of each isolation was checked as the respiratory control ratio of the sample with high resolution respirometry.

### 2.4. Western Blots

Phosphorylation of S6 ribosomal protein and AMPK in fibroblast total cell lysate was determined as a ratio between signals of phosphorylated and total protein on western blot. Antibodies for S6 ribosomal protein and phospho-S6 ribosomal protein (Ser235/236) and for AMPK*α* and phospho-AMPK*α* (Thr 172) were from Cell Signaling Technology (Danvers, MA, USA) and used in dilution 1 : 1000 in 5% bovine serum albumin at 4°C overnight. Ratio of LC3bII/LC3bI protein was determined with western blot in lysates of either intact cell or cells treated with 50 *μ*M chloroquine (Sigma-Aldrich) for 6 hours. Both bands were detected with LC3 antibody from Novus Biologicals (Cambridge, UK) diluted 1 : 1000 in 5% bovine serum albumin at 4°C overnight. Secondary antibodies conjugated with horseradish peroxidase (Sigma-Aldrich) were used in dilution 1 : 5000 in 5% bovine serum albumin at 25°C for 1 hour. The membranes were then incubated with SuperSignal West Pico Chemiluminescent Substrate (Thermo Fisher Scientific, Waltham, MA, USA) for 5 minutes and the intensity of the signal was measured with chemiluminescent detector LAS-1000 (Fujifilm). Densitometric analyses of the signals were performed with ImageJ software (public domain freeware).

### 2.5. Gene Expression and Mitochondrial DNA Copy Number

Total liver and fibroblast DNA was isolated with solvent extraction as described previously [27]. Total liver RNA was isolated with Trizol reagent (Life Technologies, Carlsbad, CA, USA) according to manufacturer's protocol and subjected to cDNA preparation with Maxima reverse transcriptase (Thermo Fisher Scientific) using oligo dT primers. Mitochondrial DNA copy number, a marker of mitochondrial abundance, was determined with real time PCR as described previously [28]. Expressions of mRNA for mitochondrial transcription factor A (TFAM) and mitochondrial DNA polymerase gamma (POLG), prototypical markers of mitochondrial biogenesis and PGC1*α* activity, and GAPDH as a reference were determined with qRT-PCR with the following set of primers:

 TFAM fwd-TTCCAGGGGGCTAAGGATGA
 TFAMre-CACACTGCGACGGATGAGAT
 POLGfwd-CCTCGTTATCGGAGGCCCAAT
 POLGre-GGCTGGTCCAAGAGTAACGC
 GAPDHfwd-ATGTCAGATCCACAACGGATACA
 GAPDHre-AACTCCCTCAAGATTGTCAGCAA.

### 2.6. Production of H_2_O_2_ from Fibroblasts and Mitochondria

The production of H_2_O_2_ from intact fibroblasts (10^6^ cells/mL) in PBS supplemented with 5 mM glucose and 3 mM glutamine and from the isolated mitochondria (0.2 mg/mL) in the isolation buffer with 10 mM glutamate, 2 mM malate, and 10 mM succinate was determined with spectrofluorimetry (RF-5301PC, Shimadzu, Kyoto, Japan) using the oxidation of the fluorogenic indicator Amplex Red (Life Technologies) in the presence of horseradish peroxidase (HRP, Sigma-Aldrich) as described previously [29].

### 2.7. Mitochondrial Matrix ROS Production in Fibroblasts

The production of ROS into the mitochondrial matrix was determined as the slope of a time-dependent increase in the fluorescence of a selective MitoSOX probe (10 *μ*M; Life Technologies) in live fibroblasts treated with/without 5 mM l-lactate by FACS analysis (BD LSRII, BD Biosciences, CA, USA) as described previously [26].

### 2.8. Determination of GSSG/GSH

To estimate the changes in global cellular redox state, the ratio of oxidized/total glutathione (GSSG/total GSH) was determined from cell lysates by capillary electrophoresis (Agilent 7100, Agilent Technologies, Santa Clara, CA, USA) equipped with a polyimide-coated fused silica capillary (68 cm × 50 *μ*m). Cells were gently scraped and collected in centrifugation tube with its culture medium. Tube was centrifuged at 300 ×g for 5 minutes; cell pellets were quickly washed with ice-cold phosphate-buffered saline and immediately lysed in acetonitrile/water (3 : 2, v/v). Lysates were centrifuged at 20,000 ×g for 20 min at 4°C. Supernatant containing glutathione was collected, snap frozen, and stored in liquid nitrogen up to 6 hours before the analysis. Separation was done in 40 mM phosphate buffer pH 7.0, voltage was set at 30 kV, and absorbance was measured at 195 nm. Reduced and oxidized glutathione were detected as separate peaks and identified by a comparison with analytical grade standards (Sigma-Aldrich). GSSG percentage was calculated from the areas of the individual peaks of GSH and GSSG as described previously [26].

### 2.9. Analyses of Metabolites

Levels of lactate, malate, citrate, fumarate, 2-oxoglutarate, and 2-hydroxyglutarate in fibroblasts were determined with gas chromatography-mass spectrometry (GC 6890N, MD 5973, Agilent Technologies). Cells were gently scraped and collected in centrifugation tube with its culture medium. Tube was centrifuged at 300 ×g for 5 minutes, cell pellets were quickly washed with ice-cold phosphate-buffered saline and immediately frozen and stored at −80°C up to 1 week. For one analysis, 10^6^ cells, collected from 60 cm^2^ of confluent culture plate, were employed. Internal standard oxalate was then added and the samples were extracted with water/methanol/chloroform (1 : 1 : 2, v/v/v) and centrifuged at 1000 ×g for 10 min. The upper polar phase was transferred into glass vial and lyophilized. The analytes were derivatized with pyridine/N-(trimethylsilyl)acetamide/chlorotrimethylsilane (9 : 3 : 1, v/v/v, all from Sigma-Aldrich) at 65°C for 1 hour. Derivatized samples were directly injected into gas chromatography-mass spectrometry. During the run, initial temperature 100°C of column was used and held for 1 minute, then column temperature was increased to 180°C (linear increase 10°C per minute took 8 minutes) and held until the end of an analysis which total time was 10 minutes. Retention times of the selected metabolites were confirmed with analytical grade standards (Sigma-Aldrich) and detectable specific mass ions were chosen according to their mass spectra. Total weights (ng per 10^6^ cells) of lactate (*m/z* 219), malate (*m/z* 335), citrate (*m/z* 273), fumarate (*m/z* 245), 2-oxoglutarate (347), and 2-hydroxyglutarate (349) were calculated from ratio between the respective ions and the internal standard ion (*m/z* 190) after the alignment with standard curves prepared from the respective analytical grade standards (Sigma-Aldrich).

### 2.10. High Resolution Respirometry

To assess the function of mitochondrial respiratory chain, the respiration of the cells (10^6^ cells/mL) in the complete media and the isolated mitochondria (0.2 mg/mL) in the isolation buffer with 10 mM glutamate, 2 mM malate, and 10 mM succinate was measured with the Oroboros O2k oxygraph (Oroboros Instruments, Innsbruck, Austria) as described previously [26]. Maximal respiration was measured after uncoupling of mitochondria with carbonyl cyanide-*4*-(trifluoromethoxy)phenylhydrazone (FCCP, Sigma-Aldrich).

### 2.11. Confocal Microscopy

An overall shape and integrity of a fibroblast mitochondrial network were assessed after staining of mitochondria in the live fibroblasts with 10 *μ*M tetramethylrhodamine (TMRE, Life Technologies), or after staining with 10 *μ*M MitoTracker Orange (Life Technologies). The density of cristae was assessed in fixed cells (with 4% formaldehyde/0.04% glutaraldehyde) after staining with antibody against mitofilin (1 : 1000 in 5% donkey serum, Cell Signaling Technology) and with the secondary antibody conjugated with fluorescent dye Alexa Fluor 488 (Life Technologies). The microscopic inspection was performed with confocal microscopy (Leica TSC SP2, Microsystems, Wetzlar, Germany).

### 2.12. Statistical Analyses

Statistical analyses were performed in the SigmaPlot software (Systat software, San Jose, CA, USA) and graphs were created in the MS Excel software (Microsoft, Redmond, WA, USA). Data are expressed as mean ± SD. Statistical significance of the results was tested with Student's *t*-test (for normally distributed data), Mann-Whitney *U* Rank Sum test (for skewed data), or Student-Newman-Keuls ANOVA (for multiple comparisons). Differences were considered statistically significant when *P* values were less than 0.05.

## 3. Results and Discussion

### 3.1. L-Lactate Causes H_2_O_2_ Burst and a Mild Inhibition of Respiration

**Figure 1:**
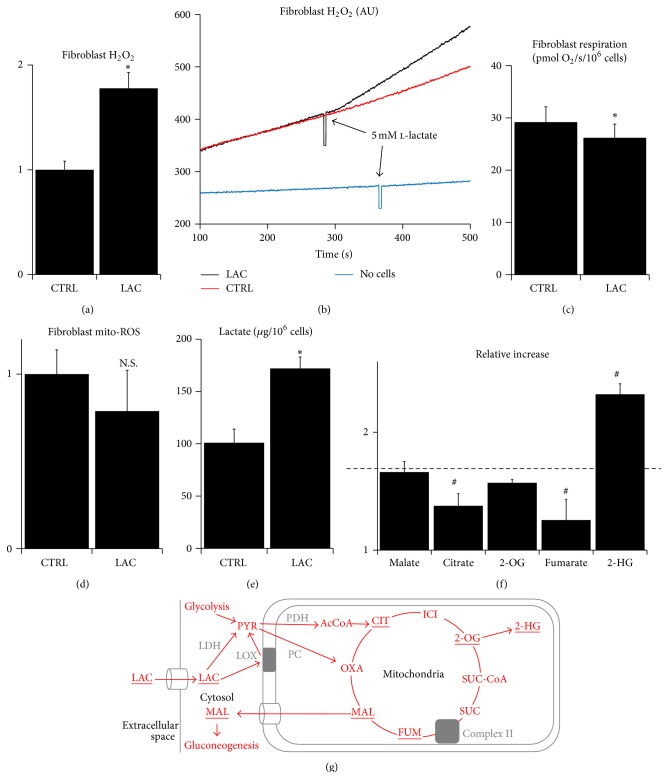
The effect of l-lactate on respiration, H_2_O_2_ production, and metabolism of skin fibroblasts. (a) Relative production of H_2_O_2_ from intact fibroblasts measured with Amplex Red before (CTRL) and after (LAC) the addition of 5 mM l-lactate. (b) Representative spectrofluorimetric track from the same measurement. (c) Respiration of intact fibroblasts in complete media before (CTRL) and after (LAC) addition of 5 mM l-lactate. (d) Relative production of ROS (superoxide and peroxonitrite) in mitochondrial matrix of intact fibroblasts measured with MitoSOX before (CTRL) and after (LAC) the addition of 5 mM l-lactate. (e) Intracellular concentrations of l-lactate in fibroblasts cultured in complete media before (CTRL) and after (LAC) addition of 5 mM l-lactate. (f) Relative increase in the intracellular levels of selected metabolites after the addition of 5 mM l-lactate (dashed line: value for l-lactate). (g) Scheme of l-lactate utilization in the cell. LAC: lactate; PYR: pyruvate; CIT: citrate; ICI: isocitrate; 2-OG: 2-oxoglutarate; 2-HG: 2-hydroxyglutarate; SUC: succinate; FUM: fumarate; MAL: malate; OXA: oxaloacetate; Ac: acetyl; LDH: lactate dehydrogenase; LOX: lactate oxidase; PC: pyruvate carboxylase; PDH: pyruvate dehydrogenase (^∗^significantly different from CTRL, *P* < 0.05; ^#^significantly different from l-lactate, *P* < 0.05).

The hallmark of mitohormesis is a mild inhibition of mitochondrial respiratory chain associated with a moderate elevation of mitochondrial ROS production [4, 5, 7, 8]. It has been previously reported that 20 mM l-lactate elevates the ROS levels in L6 smooth muscle cell lines [17]. However, the serum levels of l-lactate during aerobic exercise usually do not exceed 5 mM [30]. Therefore, we tested whether a physiologically more relevant 5 mM l-lactate dose could cause a mitochondrial ROS burst in skin fibroblasts. Indeed, the addition of 5 mM l-lactate to skin fibroblasts supplemented with 5 mM glucose and 3 mM glutamine resulted in an immediate 75% increase in cellular H_2_O_2_ production (Figures 1(a) and 1(b)). This effect was associated with a mild but significant suppression of cellular respiration (Figure 1(c)). Interestingly, the release of ROS (superoxide and peroxonitrite) into mitochondrial matrix was not influenced by l-lactate (Figure 1(d)). We further performed a metabolic profiling to characterize the impact of l-lactate treatment on the mitochondrial metabolism. Supplementation of the cells with 5 mM l-lactate increased the intracellular l-lactate level by only 70% (Figure 1(e)) which is in line with the finding that fibroblasts in normoxia produce significant amounts of l-lactate [31]. The intracellular levels of Krebs cycle components malate, citrate, and 2-oxoglutarate (Figure 1(f)) were elevated to the similar extent as l-lactate, suggesting that l-lactate stimulates a repletion of intermediary metabolites presumably through the increased synthesis of oxaloacetate/malate from l-lactate-derived pyruvate (Figure 1(g)) [32]. However, the elevation of intracellular fumarate (Figure 1(f)) was very slight, while the elevation of 2-hydroxyglutarate (Figure 1(f)) was much greater than that of the other metabolites. Since 2-hydroxyglutarate is a marker of the Krebs cycle stalling due to the impaired respiratory chain activity [33], and since the synthesis of fumarate from succinate requires the activity of the respiratory chain Complex II [33], we suggest that these changes further support the mild inhibition of the mitochondrial respiratory chain activity by l-lactate.

**Figure 2:**
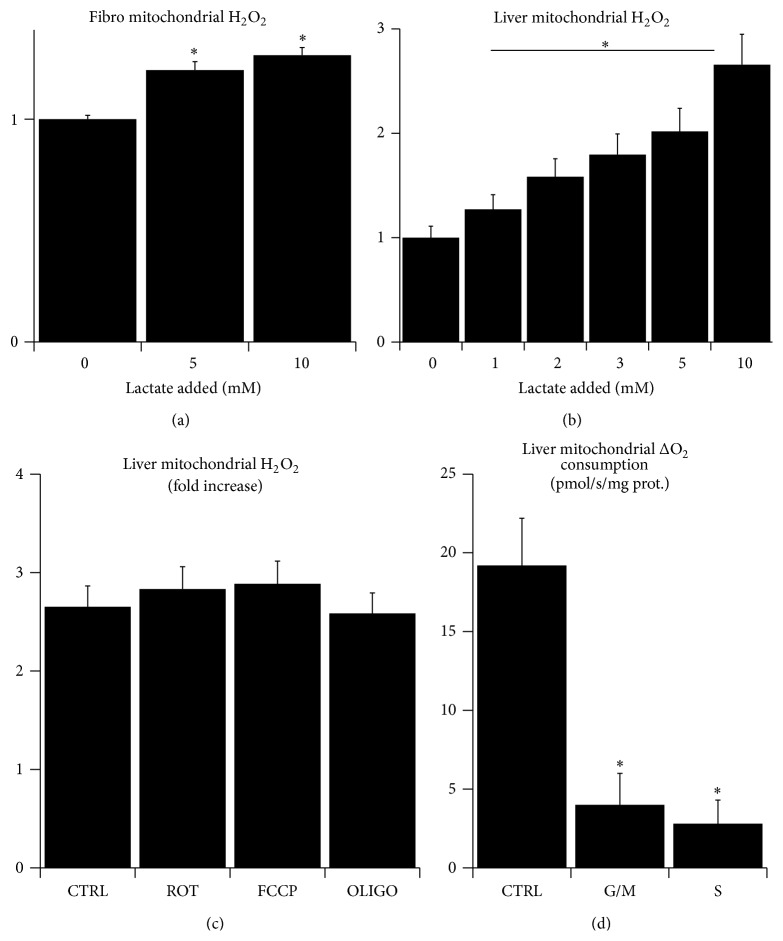
The effect of l-lactate on respiration and H_2_O_2_ production of isolated mitochondria. (a) Relative production of H_2_O_2_ from isolated fibroblasts mitochondria respiring on 10 mM glutamate, 1 mM malate, and 10 mM succinate measured with Amplex Red after the addition of 0–10 mM l-lactate. (b) Relative production of H_2_O_2_ from isolated liver mitochondria respiring on 10 mM glutamate, 1 mM malate, and 10 mM succinate measured with Amplex Red after the addition of 0–10 mM l-lactate. (c) Relative increase in production of H_2_O_2_ from isolated liver mitochondria without substrates (CTRL), pretreated with 10 *μ*M rotenone (ROT), 10 *μ*M FCCP (FCCP), or 2 mg/L oligomycin (OLIGO) measured by Amplex Red after addition of 5 mM l-lactate. (d) Increase in O_2_ consumption of isolated mitochondria without substrates (CTRL), respiring on 10 mM glutamate/1 mM malate (G/M) or respiring on 10 mM succinate (S) after addition of 5 mM l-lactate. (^∗^significantly different from CTRL or from 0 mM l-lactate; *P* < 0.05).

In the next step, we aimed to further characterize the source of H_2_O_2_ burst upon l-lactate treatment. The addition of 5 or 10 mM l-lactate to isolated fibroblast mitochondria resulted in the significant increase in H_2_O_2_ production (Figure 2(a)) suggesting that the source of H_2_O_2_ is localized to mitochondria. Since fibroblasts gave only a small yield of low quality mitochondria, we performed the next set of experiments with mitochondria isolated from rat liver. The treatment of liver mitochondria respiring on glutamate/malate/succinate with 1–10 mM l-lactate showed a dose-dependent increase in H_2_O_2_ production (Figure 2(b), *P* for trend <0.001). However, the release of H_2_O_2_ neither was dependent on the presence of substrates other than lactate, nor was affected by the inhibition of mitochondrial Complex I with rotenone, inhibition of F_0_F_1_ ATP synthase by oligomycin, or uncoupling of mitochondria with FCCP (Figure 2(c)). These results suggest that the source of l-lactate-driven H_2_O_2_ burst is the previously reported putative mitochondrial l-lactate oxidase localized in the intermembrane space and oxidizing l-lactate to pyruvate [34]. In addition, we tested whether l-lactate causes the mild inhibition of respiration also in isolated mitochondria. Interestingly, while the addition of 5 mM l-lactate to the isolated liver mitochondria without other substrates resulted in a net increase of oxygen consumption as a result of l-lactate oxidase activity, the addition of 5 mM l-lactate to the mitochondria respiring either on glutamate/malate or on succinate did not influence the oxygen consumption (Figure 2(d)). This effect could be explained by the mild inhibition of respiration which outweighs the l-lactate-oxidase-derived oxygen consumption.

Taken together, these data show for the first time that the elevation of l-lactate to the levels observed during exercise causes a mild inhibition of mitochondrial respiratory chain associated with the enhanced production of H_2_O_2_.

### 3.2. L-Lactate Activates AMPK and Mitochondrial Biogenesis

**Figure 3:**
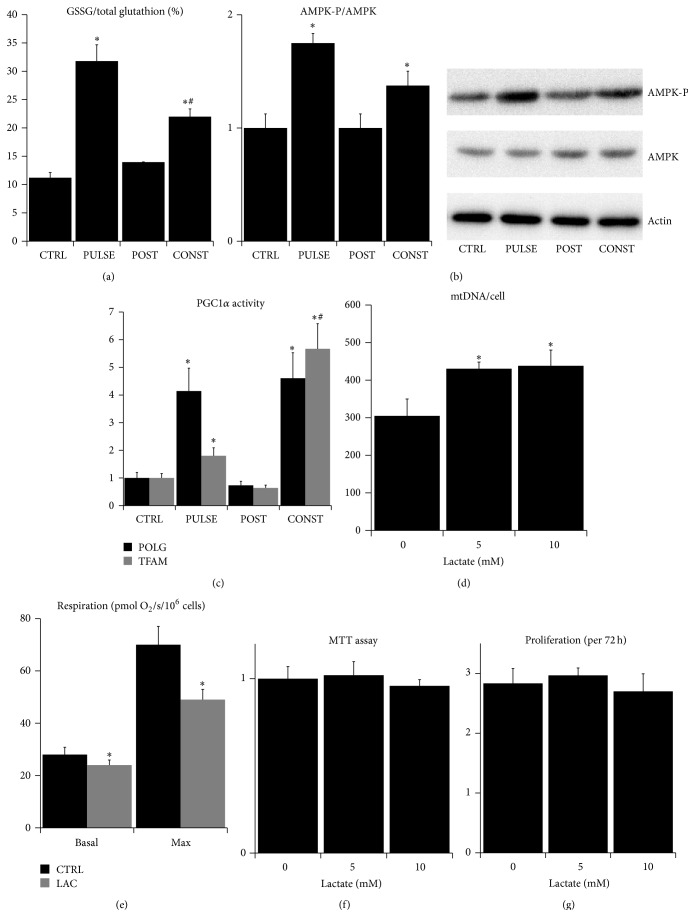
The response of fibroblasts intracellular signaling to l-lactate treatment. (a) Percentage of oxidized (GSSG)/total intracellular glutathione in fibroblasts before (CTRL) and 1 hour after addition of 5 mM l-lactate (PULSE), 24 hours after l-lactate withdrawal (POST), and 3 days after addition of 5 mM l-lactate (CONST). (b) Relative ratio of phosphorylated/total AMPK in fibroblasts before (CTRL) and 1 hour after addition of 5 mM l-lactate (PULSE), 1 hour after l-lactate withdrawal (POST), and 3 days after addition of 5 mM l-lactate (CONST). (c) Relative expression (normalized to GAPDH mRNA) of TFAM and POLG mRNAs as a measure of PGC1*α* activity in fibroblasts before (CTRL) and 4 hours after addition of 5 mM l-lactate (PULSE), 24 hours after l-lactate withdrawal (POST), and 3 days after addition of 5 mM l-lactate (CONST). (d) mtDNA copy number per cell as a measure of mitochondrial abundance in fibroblasts treated with 0–10 mM l-lactate for 3 days. (e) Basal and maximal (after uncoupling with FCCP) respiration of fibroblasts in complete media without treatment (CTRL) or 3 days after addition of 5 mM l-lactate (LAC). (f) Relative signal from MTT assay performed in fibroblasts treated with 0–10 mM l-lactate for 3 days. (g) Proliferation of fibroblasts treated with 0–10 mM l-lactate for 3 days measured by direct counting of cells negative for Trypan blue inclusion. (^∗^significantly different from CTRL; *P* < 0.05; ^#^significantly different from PULSE; *P* < 0.05).

Next, we studied whether the l-lactate treatment could activate the signaling pathways responsible for the beneficial effects of mitohormesis. The previous reports showed that the effect of mitohormesis is dependent on altered redox signaling and on the activation of AMPK which is the master regulator of cellular stress response [7–9, 35]. We found that the addition of 5 mM l-lactate to skin fibroblasts significantly increased the proportion of oxidized intracellular glutathione, suggesting the presence of oxidative stress and the shift in the cellular redox state towards oxidation (Figure 3(a)). The relative amount of the oxidized glutathione returns to the normal upon l-lactate withdrawal, while the long-term (3 days) incubation of the fibroblasts with l-lactate maintained the percentage of oxidized glutathione higher, but not to the same extent as the short-term treatment, suggesting a partial compensation of the oxidative stress with the antioxidant system (Figure 3(a)). Importantly, addition of 5 mM l-lactate to fibroblasts significantly increased the phosphorylation/activation of AMPK (Figure 3(b)). While l-lactate withdrawal returned the AMPK phosphorylation to the control level, the long-term (3 days) incubation with l-lactate maintained the AMPK phosphorylation increased, although to a lesser extent (Figure 3(b)). AMPK is also a known activator of PGC1*α*, a transcriptional coactivator and the master regulator of mitochondrial biogenesis [36]. PGC1*α* is also indispensable for the beneficial effects of physical exercise, and its overexpression is associated with the protection against aging [37, 38]. Therefore, we measured the expression of two PGC1*α*-governed genes responsible for the mitochondrial biogenesis: the mitochondrial transcription factor A (TFAM) and the mitochondrial DNA polymerase gamma (POLG). The levels of TFAM and POLG mRNAs were significantly increased upon the l-lactate treatment and the increase was more pronounced during the long-term (3 days) incubation, while the l-lactate withdrawal returned the expression to the control levels (Figure 3(c)). We further tested whether the increased expression of PGC1*α*-governed genes is actually translated to the higher mitochondrial abundance. Indeed, we found that the long-term (3 days) incubation with either 5 mM or 10 mM l-lactate resulted in the significantly increased mitochondrial DNA (mtDNA) copy number per cell (Figure 3(d)). In contrast, the same incubation significantly decreased the basal and the maximal respirations of the fibroblasts (Figure 3(e)) suggesting that the inhibition of mitochondrial respiratory chain with l-lactate is even more pronounced during the long-term incubation. These results suggest that incubation with 5 mM l-lactate represents a significant oxidative and energetic stress. Therefore, we tested whether the long-term (3 days) incubation with either 5 mM or 10 mM l-lactate compromises the viability of fibroblasts. However, we found that neither MTT assay (Figure 3(f)) nor cell proliferation (Figure 3(g)) was altered by l-lactate treatment, suggesting that the stress derived from the presence of l-lactate is not detrimental to fibroblasts.

Taken together, we have demonstrated that even the short-term treatment of fibroblasts with l-lactate levels observed during exercise activates the signaling pathways involved in mitohormesis, that is, the shift in the intracellular redox state and the phosphorylation of AMPK, and that this signaling is associated with the enhanced transcriptional activity of PGC1*α* but not with increased mitochondrial respiratory capacity.

### 3.3. Intermittent Treatment with  l-Lactate Protects against Mitochondrial Dysfunction

**Figure 4:**
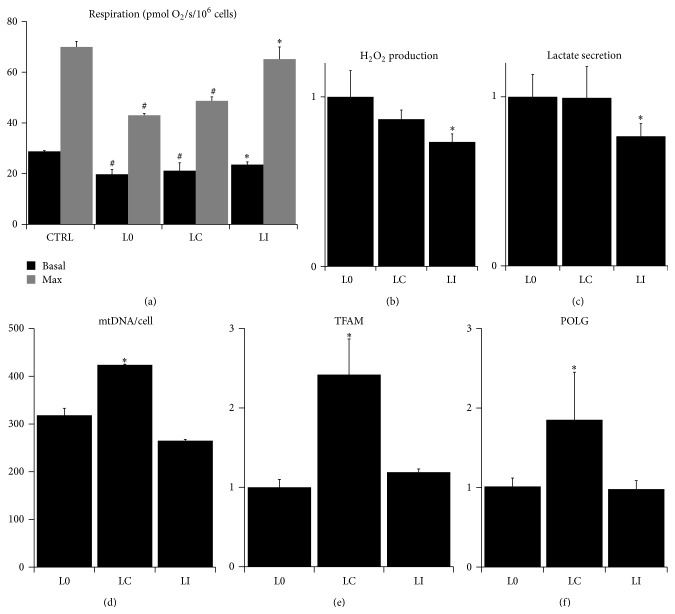
Markers of mitochondrial dysfunction in high-passage fibroblasts. (a) Basal (black bars) and maximal (grey bars; after uncoupling with FCCP) respiration of fibroblasts in complete media. (b) Relative production of H_2_O_2_ from fibroblasts measured by Amplex Red. (c) Relative rate of secretion of lactate into culture media from fibroblasts. (d) mtDNA copy number per cell as a measure of mitochondrial abundance in fibroblasts. Relative expression (normalized to GAPDH mRNA) of (e) TFAM and (f) POLG mRNAs as a measure of PGC1*α* activity in fibroblasts (control fibroblasts: passage 2 (CTRL); control fibroblasts: passage 18 (L0); fibroblasts cultured in constant 5 mM l-lactate: passage 18 (LC); and fibroblasts exposed to twice a week pulses of 5 mM l-lactate: passage 18 (LI); ^∗^significantly different from L0; *P* < 0.05; ^#^significantly different from CTRL; *P* < 0.05).

**Figure 5:**
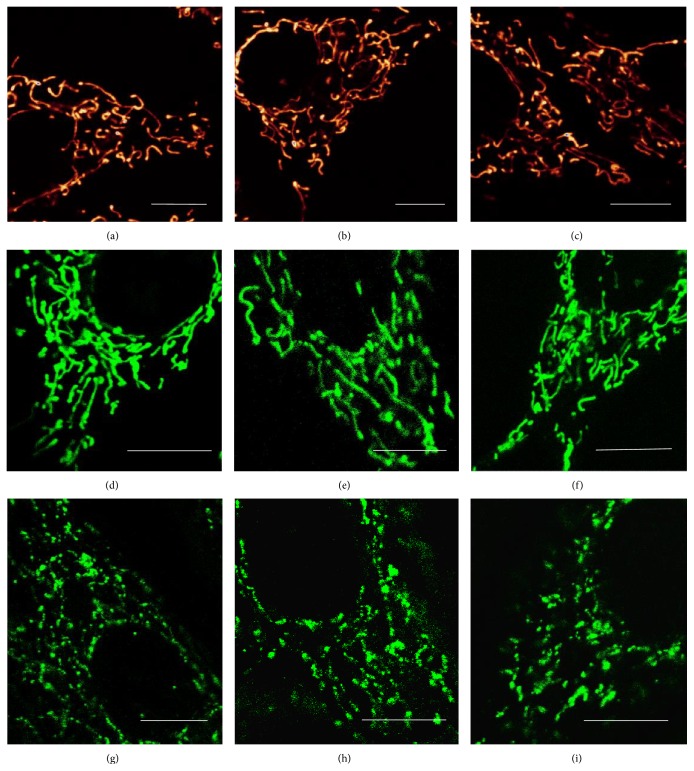
Shape and integrity of mitochondrial reticulum in high-passage fibroblasts. Mitochondrial reticulum of live (a) L0, (b) LC, and (c) LI fibroblasts was stained with 10 *μ*M tetramethylrhodamine (TMRE) and its overall shape was inspected with confocal microscopy. Mitochondrial reticulum of live (d) L0, (e) LC, and (f) LI fibroblasts was stained with 10 *μ*M MitoTracker Orange and its overall shape was inspected with confocal microscopy. Mitochondrial cristae-shaping protein mitofilin was visualized in fixed (g) L0, (h) LC, and (i) LI fibroblasts with antibody and its overall distribution was inspected with confocal microscopy (control fibroblasts: passage 18 (L0); fibroblasts cultured in constant 5 mM l-lactate: passage 18 (LC); and fibroblasts exposed to twice a week pulses of 5 mM l-lactate: passage 18 (LI); bar = 10 *μ*m).

The mitohormesis and PGC1*α* upregulation have been shown to protect against mitochondrial dysfunction, one of the hallmarks of aging [35, 37]. Mitochondrial dysfunction is caused by mutations in mtDNA and accumulation of misfolded proteins in mitochondria which compromise the proper function of the respiratory chain [39]. Mitochondrial dysfunction is usually characterized by the decrease in mitochondrial respiration and the increase in the secretion of l-lactate out of the cell. This effect could be further accompanied by the increased leak of ROS from mitochondrial respiratory chain, diminished mitochondrial membrane potential, or the presence of altered mitochondrial shape and ultrastructure [40, 41]. Since l-lactate treatment appeared to trigger mitohormesis and induce PGC1*α* activity, we tested whether it could prevent the onset of mitochondrial dysfunction in the skin fibroblasts undergoing replicative aging. The replicative aging of primary fibroblasts in the culture is a widely used* in vitro* model of mammalian aging [42]. In our settings, fibroblasts were cultured either without any l-lactate added (L0), or in a constant 5 mM l-lactate (LC), or exposed to intermittent (twice a week) 5 mM l-lactate (LI). In the first 18 passages, L0, LC, and LI fibroblasts showed a constant and similar rate of proliferation and respiration independently of treatment group or passage number. However, in passage 18 and above, we observed a sharp and significant attenuation of the basal and maximal cellular respiration in L0 and LC, but not in the LI fibroblasts (Figure 4(a)). This effect was associated with a significantly lower rate of the cellular H_2_O_2_ release in LI fibroblasts compared to L0 and LC (Figure 4(b)). Moreover, the extracellular release of l-lactate was significantly lower in LI fibroblasts compared to L0 and LC (Figure 4(c)). Importantly this effect was not dependent on mitochondrial abundance, since the mtDNA copy number per cell (Figure 4(d)) and the expression of TFAM (Figure 4(e)) and POLG (Figure 4(f)) were not different between L0 and LI fibroblasts and were even higher in LC fibroblasts. In addition, confocal microscopy showed no difference between L0, LC, and LI fibroblasts in terms of mitochondrial membrane potential (Figures 5(a), 5(b), and 5(c)), shape of the mitochondrial reticulum (Figures 5(d), 5(e), and 5(f)), and distribution of the mitochondrial protein mitofilin which shapes mitochondrial cristae (Figures 5(g), 5(h), and 5(i)).

These findings suggest that fibroblasts in passage 18 and above develop a kind of mitochondrial dysfunction which is characterized by the impairment of the respiratory chain function but is not associated with changes in mitochondrial biogenesis or morphology. Importantly, the mitochondrial dysfunction is prevented by intermittent but not constant exposure to l-lactate. The mitochondrial dysfunction associated with aging is believed to result from a chronological accumulation of oxidative damage to mtDNA due to its proximity to ROS released from the respiratory chain [2, 3]. The intermittent activation of protective and quality control mechanisms through AMPK, PGC1*α*, and redox signaling in LI cells thus seems to be sufficient to protect mtDNA against the damage. Therefore, mild intermittent oxidative stress and inhibition of mitochondrial respiratory chain associated with l-lactate exposure could protect against deleterious oxidative damage and mitochondrial dysfunction associated with aging. In contrast, the lack of protection in LC fibroblasts could be attributed to the constant oxidative stress which outweighs the effect of protective mechanisms thus neutralizing the beneficial effects of mitohormesis.

### 3.4. Treatment with  l-Lactate Affects mTOR, Senescence, and Autophagy

**Figure 6:**
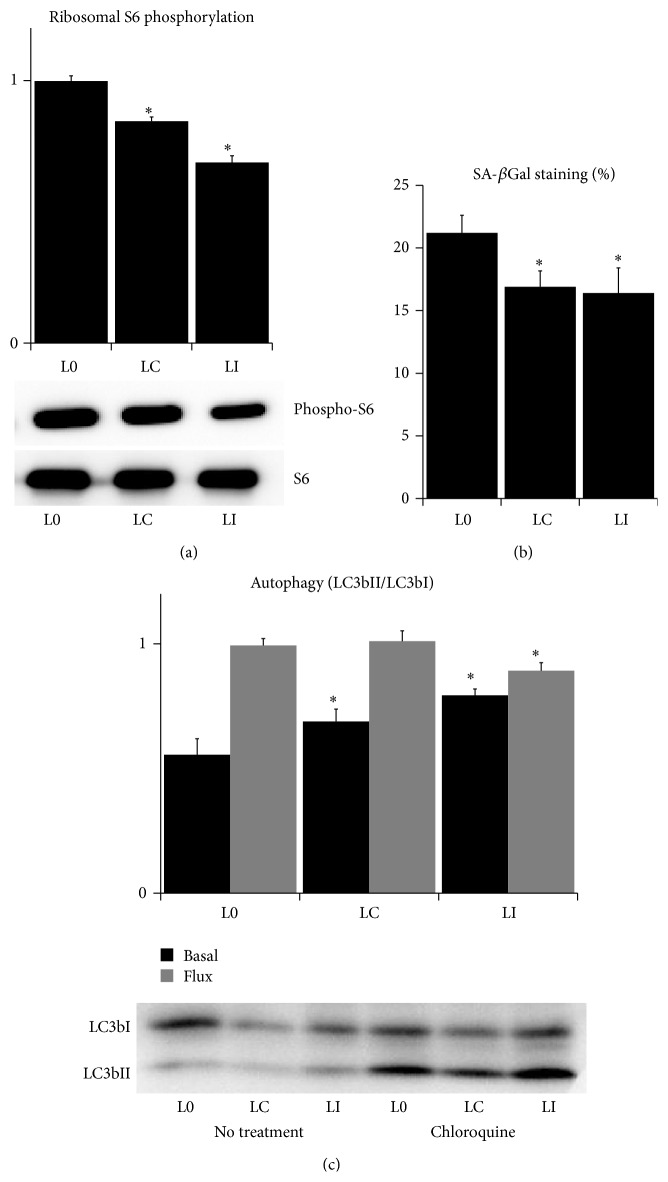
mTOR, senescence, and autophagy in high-passage fibroblasts. (a) Relative ratio of phosphorylated/total S6 ribosomal protein in aged fibroblasts. (b) Percentage of aged fibroblasts positive for senescence-associated *β*-galactosidase staining. (c) Ratio of LC3bII/LC3bI isoforms in intact aged fibroblasts (basal) or after 6-hour treatment with 50 *μ*M chloroquine (flux) (control fibroblasts: passage 18 (L0); fibroblasts cultured in constant 5 mM l-lactate: passage 18 (LC); and fibroblasts exposed to twice a week pulses of 5 mM l-lactate: passage 18 (LI); ^∗^significantly different from L0; *P* < 0.05).

The presence of mitochondrial dysfunction, altered redox signaling, and AMPK activity could affect also other signaling pathways involved in aging. One of the most robust molecular determinants of aging is the mechanistic target of rapamycin complex 1 (mTORC1), the master regulator of cell growth and proliferation whose activity is inversely associated with the rate of aging [43]. Its activity is inhibited by the AMPK phosphorylation [43] but increased by shifting the cellular redox status towards the oxidation [44]. Therefore, we measured the extent of phosphorylation of S6 ribosomal protein, a marker of mTORC1 activity [43], in L0, LC, and LI fibroblasts in passage 18 and above. The phosphorylation of S6 ribosomal protein was significantly lower in LI cells compared to L0 and LC (Figure 6(a)). However, we observed no changes in the cell proliferation between L0, LC, and LI cells during the whole campaign (data not shown), suggesting that the observed changes in mTORC1 activity were insufficient to influence the cell proliferation, or that the decreased mTORC1 activity is compensated by the better bioenergetics in the LI cells compared to the L0 and LC. The mTORC1 activity and the mitochondrial ROS release also promote the onset of cellular senescence, a distinct cell phenotype which is highly abundant in aged tissues and contributes to aging [43, 45]. The percentage of cells positive for the senescence-associated *β*-galactosidase staining was significantly lower in LI and LC fibroblasts compared to L0 (Figure 6(b)). Finally, mTORC1 is the inhibitor of autophagy [43], a quality control process which is implicated in the protection against mitochondrial dysfunction and aging [46]. We measured the rate of autophagophores formation as a ratio of LC3bII/LC3bI proteins and the autophagic flux as a relative increase of this ratio after the inhibition of the autophagosome-lysosome fusion with the chloroquine [47]. We found the increased formation of autophagophores in LI fibroblasts compared to L0 and LC, but the lower autophagic flux in LI fibroblasts compared to L0 and LC (Figure 6(c)). This finding could be interpreted as the increased activation of autophagy in LI fibroblasts due to the lower activity of mTORC1 which is, however, not implemented into the autophagic flux due to the higher quality of LI mitochondria and the lower oxidative damage in LI cells.

**Figure 7:**
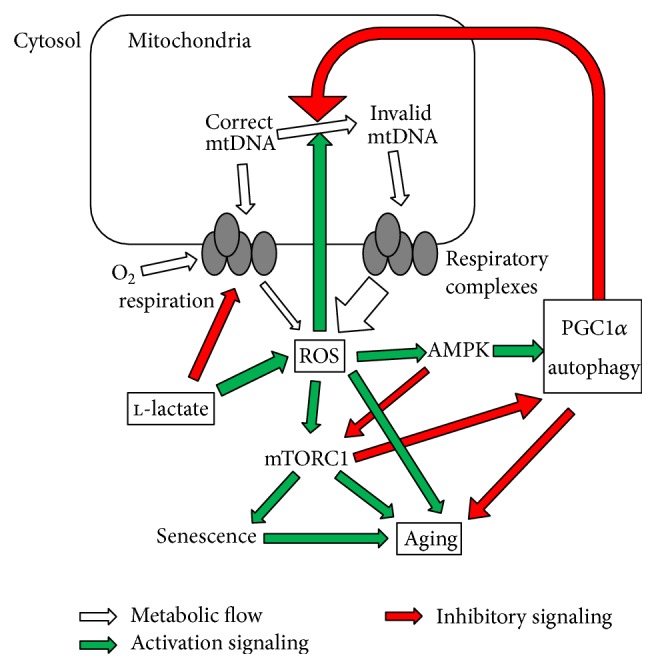
Overview of the main signaling pathways studied in this paper.

Taken together, the absence of mitochondrial dysfunction and oxidative stress in LI fibroblasts significantly attenuated the activity of mTORC1, decreased the transition to cellular senescence, and increased the activity of autophagy pathway. All these mechanisms could further contribute to the antiaging effects of intermittent l-lactate exposure (Figure 7).

## 4. Conclusions

L-lactate is a natural compound widely used in food and cosmetic industry because of its safety and excellent antimicrobial properties. Moreover, l-lactate levels in human body are intermittently elevated during physical exercise, the condition associated with the prevention of aging-associated diseases and decreased mortality. Here, we describe that intermittent treatment of skin fibroblasts with l-lactate levels occurring during exercise causes the mild inhibition of mitochondrial respiratory chain and the increased production of mitochondrial H_2_O_2_. These effects cause the intermittent shift in the intracellular redox state towards oxidation and trigger the protective and quality control signaling including the phosphorylation of AMPK and the increased transcriptional activity of PGC1*α*. The long-term intermittent application, but not the constant application, of l-lactate prevents the onset of mitochondrial dysfunction in aged skin fibroblasts, that is, the drop in respiration and the rise of ROS production. The absence of mitochondrial dysfunction in fibroblasts treated with the intermittent l-lactate is associated with the lower activity of mTORC1, the less frequent transition of cells to senescence, and the higher activity of the intracellular quality control by autophagy. In conclusion, the mitohormesis resulting from the intermittent exposure to l-lactate exhibits a potent antiaging effect at least in skin fibroblasts. These results shed a new light on the role of l-lactate in the beneficial effects of physical exercise and in cosmeceutical and nutraceutical preparations.
